# GTfold: Enabling parallel RNA secondary structure prediction on multi-core desktops

**DOI:** 10.1186/1756-0500-5-341

**Published:** 2012-07-02

**Authors:** M Shel Swenson, Joshua Anderson, Andrew Ash, Prashant Gaurav, Zsuzsanna Sükösd, David A Bader, Stephen C Harvey, Christine E Heitsch

**Affiliations:** 1 School of Mathematics, Georgia Institute of Technology, Atlanta, GA, USA; 2 College of Computing, Georgia Institute of Technology, Atlanta, GA, USA; 3 Interdisciplinary Nanoscience Center, Aarhus University, Aarhus, Denmark; 4 Department of Molecular Biology, Aarhus University, Aarhus, Denmark; 5 School of Biology, Georgia Institute of Technology, Atlanta, GA, USA

## Abstract

**Background:**

Accurate and efficient RNA secondary structure prediction remains an important open problem in computational molecular biology. Historically, advances in computing technology have enabled faster and more accurate RNA secondary structure predictions. Previous parallelized prediction programs achieved significant improvements in runtime, but their implementations were not portable from niche high-performance computers or easily accessible to most RNA researchers. With the increasing prevalence of multi-core desktop machines, a new parallel prediction program is needed to take full advantage of today’s computing technology.

**Findings:**

We present here the first implementation of RNA secondary structure prediction by thermodynamic optimization for modern multi-core computers. We show that GTfold predicts secondary structure in less time than UNAfold and RNAfold, without sacrificing accuracy, on machines with four or more cores.

**Conclusions:**

GTfold supports advances in RNA structural biology by reducing the timescales for secondary structure prediction. The difference will be particularly valuable to researchers working with lengthy RNA sequences, such as RNA viral genomes.

## Findings

Prediction algorithms based on thermodynamic models have been parallelized in the past, but for specific computer architectures that were available over two decades ago. The Vienna RNA package was originally parallelized [1] for a target architecture (an IBM RS/6000) popular in the early 1990’s. Subsequently [2,3], this work was extended to a first-generation cluster-like parallel computer (the Intel Delta) with an early message-passing framework, which allows the processors to exchange only coarse-grained information. Similarly, other researchers [4] parallelized a minimal free energy (MFE) folding algorithm for two architectures (a Cray Y-MP vector machine and MasPar MP-2 SIMD mesh) popular through the 1990’s.

Since then, parallel computing technology has changed substantially, and these previous parallel implementations are not compatible with current architectures. The current execution model includes multiple sockets of multi-core processors, deep memory hierarchies with several levels of cache, and the availability of gigabytes of memory. New parallel implementations are needed to take full advantage of these new platforms.

We present the first implementation of RNA secondary structure prediction by thermodynamic optimization for modern multi-core computers. Our motivation is to reduce the prediction time for long RNA sequences on modern parallel platforms, including contemporary laptops and desktops, because sequential algorithms still take many minutes to produce a single MFE structure. With the increasing prevalence of multi-core desktop machines, reduction in runtime will be particularly valuable to researchers working with long RNA sequences, such as RNA viral genomes.

We compare our program GTfold with two of the most widely-used sequential programs, UNAfold [5] and RNAfold [1,6] which also run on desktop machines. The purpose of our comparisons is to demonstrate to RNA researchers that GTfold’s runtime improvements are easily accessible from their desktop computer. Our program GTfold achieves the same minimum free energy (MFE) accuracy as UNAfold and RNAfold in appreciably less time on modern multi-core computers. With only four cores, GTfold folds an HIV genome, a sequence of nearly 10,000 nucleotides, in just 3.38 minutes versus the sequential running times of RNAfold (6.05 min.) and UNAfold (37.32 min.), a significant reduction in running time which further drops to below two minutes on eight or more cores.

In addition to our findings on runtime and accuracy given below, we summarize the algorithmic and implementation differences between these three software packages. These findings explain why, though they are based on the same basic model, they often produce different MFE structures. This information clarifies why the “same” programs can produce different outcomes.

### Implementation

Like UNAfold and RNAfold, we implement the classic dynamic programming algorithm for free energy minimization [7] based on the same general nearest neighbor thermodynamic model (NNTM) [8,9]. The optimal solution for a given sequence, that is the MFE and associated secondary structure, is computed from optimal solutions for smaller subproblems. The algorithm first fills various one- and two-dimensional tables, whose entries correspond to the MFE scores for different subsequences. It then computes an optimal structure for the full sequence by performing a traceback through these tables. For detailed descriptions of the data dependencies among various elements of the tables and the algorithm in general, see [7,10].

In general, these dependencies leave open various options regarding the order in which the cells of the tables are calculated. As we will explain, the order used by GTfold is ideal for enabling a fine-grained parallelism well suited for multi-core and other shared memory parallel machines. However, when run sequentially on one core, this approach results in a larger number of memory accesses compared with the best sequential approach. Hence, it allows better concurrency on multi-core machines at the expense of single-core performance.

More specifically, let _*s*1__*s*2_…_*sn*_be an RNA sequence of length *n*. The MFE score of the sequence is computed by filling a 1×*n* array whose values depend on multiple *n*×*n* “helper” arrays which are themselves interdependent. The (*i*,*j*) entries of these helper arrays, where *i*<*j*, holds the score of the minimum free energy structure for subsets of possible structures on the subsequence _*si*__*si* + 1_…_*sj*_.

**Figure 1:**
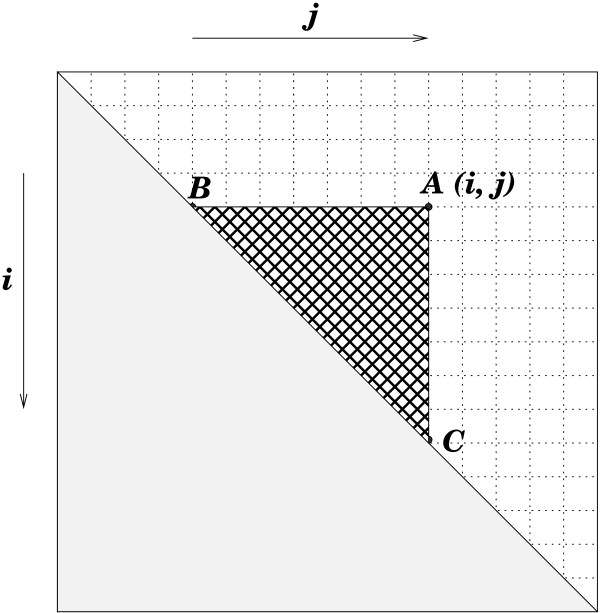
**Dependencies.** Figure 1 shows the dependencies in the dynamic programming algorithm. The (*i*,*j*) entry is dependent on the entries in the triangle (*A*,*B*,*C*).

To describe the dependencies relevant to parallelization that govern the elements of these tables, it suffices to consider a single *n*×*n*matrix and assume, for the moment, that its entries hold the optimal score for the set of possible structures on the subsequence _*si*__*si* + 1_…_*sj*_. The standard assumption of nested base pairings (i.e. lack of pseudoknots) means that the optimal structure for _*si*__*si* + 1_…_*sj*_ depends only on subsequences of the form $s_{i^{\prime \prime }}s_{i^{\prime \prime }+1}\ldots s_{j^{\prime \prime }}$, where $i<i^{\prime \prime }<j^{\prime \prime }<j$. Hence, as illustrated in Figure 1, the point (*i*,*j*) is dependent only on points $\left(i^{\prime \prime },j^{\prime \prime }\right)$ that are both below and to the left.

Consider computing the optimal score for subsequences of length *k*=*j*−*i* + 1 for some 1≤*k*≤*n*. Since these particular computations are independent of each other, they can be performed in parallel. As implemented in GTfold, the optimal structures for subsequences of length *k* are computed in parallel from *k*=1, the main diagonal, to *k*=*n*, the optimal score for the full sequence.

Other orderings, such as column-wise or row-wise, also cover the whole computation space without violating the algorithm’s dependencies. For example, RNAfold computes the dynamic programming tables in row-major order, which is cache-friendly (for programs written in C/C++, like RNAfold) and has small average memory access time, but this approach is inherently sequential.

We implement the shared memory parallelism necessary for our approach using OpenMP [11] (see Availability and requirements for details). Using OpenMP allows all threads to access and to update values in a single set of arrays.

As described above, our parallel implementation exploits a diagonal access of table entries, allowing for concurrent computation via fine-grained parallelism but incurring more cache misses (and hence, a larger average memory access time). As is often necessary when engineering parallel algorithms [12], we accept slower single processor performance, so that our code will run faster on the multi-processor machines that are becoming ubiquitous. Further implementation details can be found in Mathuriya et. al 2009 [13].

### Running time

The results shown here and in the rest of this section are based on GTfold (version 2.0), RNAfold distributed with the Vienna package (version 1.8.5), and UNAfold (version 3.8). When discussing running time, the number of cores is a parameter for a parallel algorithm so we refer to GTfold on *n* cores as GTfold(*n*). Running time comparisons are based on a complete HIV-1 genome [GenBank:K03454] of length 9,719 nucleotides. Further details on materials and methods, including command line arguments and computing architectures, are provided in Additional file 1: Appendix 1. (Recall that previous parallelized prediction programs do not run on modern multi-core machines. Because our primary interest was to evaluate the performance of GTfold on these current architectures, we did not include these previous parallelized programs in our study.)

**Figure 2:**
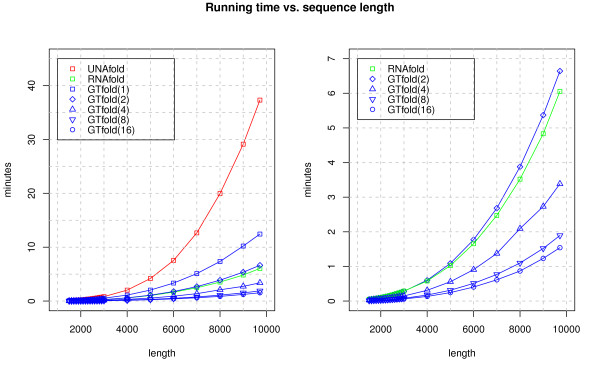
**Running time vs. sequence length.** Figure 2 shows the effect of sequence length on the running time of GTfold (run using 1, 2, 4, 8, and 16 cores), RNAfold, and UNAfold.

Our running time results for UNAfold and RNAfold versus GTfold(*n*) on *n*=1,2,4,8,16 cores are given in Figure 2. Each program predicted an MFE structure (and only that) for the HIV-1 genome, and MFE calculations also on a set of 22 complete HIV genomes of similar length (accession numbers given in Additional file 1: Appendix 1). We also document how the runtime scales with respect to length up to that point by graphing prediction times for different length subsequences of the HIV-1 genome.

We see that GTfold(*n*) is always faster than UNAfold, and significantly so for the full genome, which UNAfold computes in 37 minutes versus 3.38 minutes for GTfold(4). The relative running time of GTfold versus RNAfold depends on the number of cores used by GTfold. RNAfold computes an MFE structure for the full genome in 6.06 minutes while the running times for GTfold(*n*) are 12.44, 6.64, 3.38, 1.89, and 1.54 minutes respectively for *n*=1,2,4,8,16 cores. Hence, GTfold(1) is slower than RNAfold while the running time of GTfold(2) and RNAfold are similar (to within 10%), and GTfold on four or more cores runs in strictly less time than RNAfold.

The reproducibility of these times was confirmed by comparing UNAfold, RNAfold, and GTfold(*n*) (for *n*=1,2,4,8,16), across the full set of 22 HIV genomes (data not shown).

### Accuracy

We also demonstrate that GTfold’s reduced running time does not come at the expense of prediction accuracy, relative to UNAfold and RNAfold, as measured against ribosomal structures obtained by comparative sequence analysis [14]. We find no meaningful differences in the average prediction accuracy, which is only around 40%, of all three programs across hundreds of ribosomal sequences. This confirms that in general GTfold predicts optimal structures as accurately as UNAfold and RNAfold, while simultaneously illustrating the importance of ongoing efforts to improve RNA secondary structure prediction.

Since GTfold(*n*) always returns the same structure, we do not specify the number of cores when discussing accuracy. The reference secondary structures for 223 16S and 55 23S ribosomal sequences were downloaded from the Comparative RNA Web site [15] (database queries given in Additional file 1: Appendix 1).

**Figure 3:**
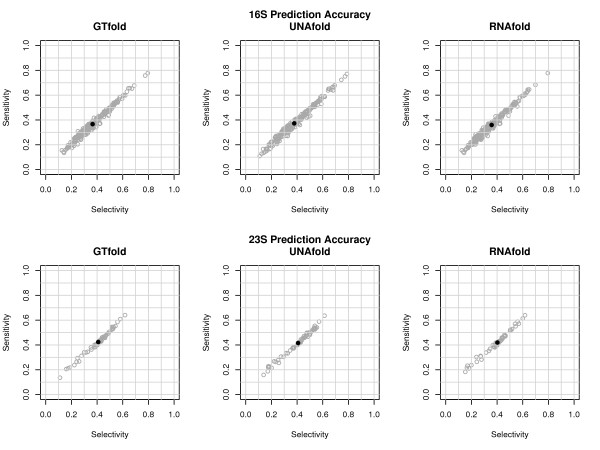
**Sensitivity vs. selectivity.** Figure 3 plots selectivity against sensitivity for GTfold, RNAfold, and UNAfold on 223 16S sequences and for 55 23S sequences. The gray circles are (selectivity, sensitivity) pairs for an individual sequence, while the black dot shows the (average selectivity, average sensitivity) for a given method on a given class of sequences.

Our prediction accuracy results for UNAfold and RNAfold versus GTfold are given in Figure 3. We assess prediction accuracy against the structures for ribosomal sequences from the Comparative RNA Web site [15] using the two measures of *sensitivity* and *selectivity* as in [16]. These values are calculated based on counting base pairs in the two structures in one of three categories. True positives (*TP*) occur in both the comparative and predicted structure, false negatives (*FN*) occur only in the comparative structure, while false positives (*FP*) occur only in the predicted structure. Gardner & Giegerich further subdivide the *FP* category to account for *compatible* base pairs, denoted *ξ*, that could exist in the comparative structure and hence “can be considered neutral with respect to algorithm accuracy.” Thus,

$$\begin{array}{cc} \text{Sensitivity} & =\frac{\text{TP}}{\text{TP}+\text{FN}}\\ \text{Selectivity} & =\frac{\text{TP}}{\text{TP}+(\text{FP}-\xi )} \end{array}$$

We find that all three programs produce structures with a wide range of sensitivity and selectivity values, with nearly indistinguishable distributions of accuracy. Each program has an average of only ∼40*%* for the 223 16S ribosomal sequences and the 55 23S ones. Given the high variance within programs (from a low of ∼10*%* to a high of ∼80*%*) versus the low variance between programs (less than 2% average difference), we find no meaningful difference in the accuracy of MFE predictions for GTfold versus UNAfold or RNAfold. (Further details are given in Additional file 2: Appendix 2.) This is certainly not to say that the three programs always predict the same optimal structure, in fact our experience indicates that the opposite is often true (particularly for UNAfold). As discussed below, this is a result of the subtle but crucial differences in the optimization criteria used by the different programs.

### Differences between programs

Finally, we address the fact that these three programs, which all implement the same basic algorithm using the same general NNTM, rarely return the same MFE structure — and in some cases return very different structures. This is due to the small but crucial differences in the thermodynamic and algorithmic details between GTfold, RNAfold, and UNAfold. The lack of robustness, or “ill-conditioning,” is a well-known aspect [17] of this particular optimization problem; slight changes in the optimization criteria can result in drastically different MFE structures.

Our analysis of GTfold’s accuracy versus RNAfold or UNAfold necessitated a detailed comparison of the different optimization criteria of the three programs. Hence, here we summarize the details that currently result in different optimal structures while noting that these differences do not have a significant effect on prediction accuracy between programs. This information should be useful to other researchers who also find the different outcomes for these “same” programs perplexing.

The optimality criteria of GTfold and RNAfold differ simply in their thermodynamic parameter values, specifically for some special cases of internal, bulge and hairpin loops and in the number of significant digits used in the entropic penalty assigned to different lengths of internal, bulge, and hairpin loops. To address this difference, GTfold includes a --rnafold option, which uses the RNAfold parameters. We have confirmed that under this option GTfold predicts the same MFE structures as RNAfold on all of our 16S and 23S sequences and on a set of 500 randomly generated sequences of length 1500.

The optimality criteria of GTfold and UNAfold differ in a number of ways. Again, there are differences in the thermodynamic parameters, but there are now also algorithmic differences, which effect the treatment of all types of loops as well as the stacked pairs. The most notable parameter differences are the treatment of G-U pairs in stacks and small symmetric internal loops. The parameters used by UNAfold differ from the Nearest Neighbor Database (NNDB) [18] values in that various substructures involving G-U pairs are assigned “infinite” energy. According to the UNAfold FAQ [19], these are instead treated as 1×1 and 2×2 internal loops, which are intended to be interpreted as base pairs. Nonetheless, it is our experience that this difference alters the optimization in small but significant ways. The algorithmic differences are four-fold: 

 1. UNAfold handles 2×3 internal loops as a special case.

 2. UNAfold considers terminal mismatches in multi-loops and external loops while GTfold instead considers pairs of 3’ and 5’ dangling ends.

 3. UNAfold performs a pre-filtering that prohibits from consideration base pairs without at least one possible neighboring pair.

 4. UNAfold and GTfold can take different traceback paths producing different optimal structures with the same MFE.

To address the parameter differences and the first three algorithmic differences, GTfold has a --unafold option. We have confirmed that under this option GTfold predicts a structure with the same MFE as UNAfold on all of our 16S and 23S sequences and on set of 500 randomly generated sequences of length 1500. We note that, due to differences in traceback, GTfold with this option does not always produce a structure identical to UNAfold despite obtaining the same minimum free energy.

### User Options

In addition to basic MFE calculation, GTfold has a number of user options. One set of options controls what information is calculated and the level of detail in the output. As is now standard, in addition to the MFE value and optimal structure, GTfold can provide a loop-by-loop energy decomposition, suboptimal structures [20] within a specified range of the MFE, and base pair probabilities via the partition function. Ongoing feature development includes performing stochastic backtracking to produce sets of structures sampled according to the Boltzmann distribution [21].

Additionally, GTfold provides several options for modifying the optimization criterion used. As with other programs, users can provide their own parameter sets for the NNTM, change the calculation of energies associated with the ends of helices in multi-loops and external loops (treatment of dangling end energies and terminal mismatches), or constrain GTfold to consider only structures that contain (or that do not contain) particular base pairs or single stranded regions. It is also possible to provide experimental data from “SHAPE” experiments (Selective 2’-Hydroxyl Acylation analyzed by Primer Extension [22]), to be used in the MFE optimization.

Finally, GTfold supports the --unafold and --rnafold options, under which it uses the same optimization criteria as UNAfold or as RNAfold, while still achieving the same running time advantages when multiple cores are available.

## Availability and requirements

GTfold is freely available as open source at gtfold.sourceforge.net. Support for parallel threads requires OpenMP (which is part of current standard C compiler packages, such as GCC). We implemented shared memory parallelism using the “omp for” directive of the OpenMP[11] interface, version 3.0 (with GCC version 4.4). Further instructions, documentation, and details on requirements are specified on the GTfold sourceforge website. 

• Project name: GTfold

• Project home page: http://gtfold.sourceforge.net/

• Operating systems: Unix, Linux, Mac OS X

• Programming language: C/C++

• Other requirements: C/C++ compiler; standard UNIX tools such as autoconf, automake, make; OpenMP support in the compiler

• License: GNU GPL v3

• Any restrictions to use by non-academics: None

## Availability of supporting data

Additional details on the sequences, software, commands, and machines used in the accuracy and running time analyses are available in Additional file 1: Appendix 1. Additional file 2: Appendix 2 provides a more detailed analysis of the relative accuracy of the three prediction methods.

## Competing interests

The authors declare that they have no competing interests.

## Authors contributions

Current version of GTfold code was written by DAB, JA, AA, PG, and ZS under the supervision of MSS, DAB, and CEH, and optimized by DAB. Numerical data was generated by PG and MSS. Paper was written by MSS and CEH, with contributions from DAB and ZS. All authors read and approved the final manuscript.

## Supplementary Material

Additional file 1 **Appendix 1**Materials and Methods. Appendix 1 gives additional details on the sequences, software, commands, and machines used in the accuracy and running time analyses. Click here for file

Additional file 2 **Appendix 2**Accuracy Comparison. Appendix 2 gives a more detailed analysis of the relative accuracy of the three prediction methods. Click here for file
