# Supplementary material for: GTfold: Enabling parallel RNA secondary structure prediction on multi-core desktops
Source: BMC Res Notes. 2012 Jul 2;5:341. doi: 10.1186/1756-0500-5-341 (PMC3748833; doi:10.1186/1756-0500-5-341)
Supplement: Additional file 1 — Appendix 1Materials and Methods. Appendix 1 gives additional details on the sequences, software, commands, and machines used in the accuracy and running time analyses. [file 1756-0500-5-341-S1.pdf]

Appendix 1: Materials and Methods

We obtained a phylogenetically diverse set of 23S and 16S sequences and corresponding secondary structures from the **Comparative RNA Website** [`http://www.rna.ccbb.utexas.edu/DAT/3B/Standard/index.php`]. To obtain the set of 16S sequences, we set the “RNA Type” to “rRNA”, the “RNA Class” to “16S”, and the “Secondary Structure” to “without” (where this final option limits the search to structures *without* pseudo knots). We then filtered the sequences (and accompanying secondary structures) to exclude any sequences containing ambiguous bases. This resulted in a set of 223 16S sequences and their structures. Using the same procedure, replacing “16S” with “23S” in the “RNA Type” field, we obtained 55 23S sequences and structures.

The GenBank accession numbers of the 22 HIV-1 genomes are [K03454, U54771, AB220944, AY271690, AF414006, AF064699, AY535659, AB286851, AJ866553, AJ291718, AF385936, AM851091, AB253421, AB253429, K03455, DQ373066, AF103818, AF084936, AY612637, AJ006022, L20571, AJ302647].

Running time comparisons are based on the time taken by the particular program in each software package that computes and returns a single MFE structure, the default for GTfold and RNAfold, and *hybrid-ss-min* for UNAfold. The specific commands used were

GTfold: `gtfold -t` *n file*

UNAfold: `hybrid-ss-min -s DAT` *file*

RNAfold: `RNAfold <` *file*

Where *file* is the input sequence file, and n is the number of cores used by GTfold.

The running time experiments are performed on an Intel(R) Xeon(R) CPU E5520 at 2.27 GHz with an x86_64 architecture, with 16 CPU's, 2 CPU sockets, 4 cores per socket and 2 threads per core, and with a L1d cache with 32K, a L1i cache with 32K, a L2 cache with 256K, and a L3 cache with 8192K.
