# Supplementary material for: GTfold: Enabling parallel RNA secondary structure prediction on multi-core desktops
Source: BMC Res Notes. 2012 Jul 2;5:341. doi: 10.1186/1756-0500-5-341 (PMC3748833; doi:10.1186/1756-0500-5-341)
Supplement: Additional file 2 — Appendix 2Accuracy Comparison. Appendix 2 gives a more detailed analysis of the relative accuracy of the three prediction methods. [file 1756-0500-5-341-S2.pdf]

Appendix 2: Accuracy Comparison

Due to the large number of data points and the correlation between the accuracy levels of the three programs, even small differences in mean accuracy are statistically significant (Table 1). However, the statistical significance does not reflect the practical significance (effect size) of the accuracy differences between these three methods. In particular, these differences do not predict which program will produce a more accurate structure for any particular sequence. For example, the difference in average accuracy on the set of 16S sequences favors UNAfold over GTfold. However, GTfold actually obtains better sensitivity on 102, and better selectivity on 87, of those 223 structures. Conversely, on the set of 23S structures where the average accuracy favors GTfold, UNAfold obtains better sensitivity on 20, and better selectivity on 22, of those 55 structures.

Table 1: Prediction accuracy: Mean sensitivity and selectivity of GTfold, UNAfold, and RNAfold over 223 16S sequences and 55 23S sequences. UNAfold and RNAfold mean accuracies marked with *, ** and *** are significantly different than GTfold accuracy, with p-values less than 0.05, 0.01 and 0.005 respectively.

	23S		16S	
	sensitivity	selectivity	sensitivity	selectivity
GTfold	0.4237	0.4091	0.3661	0.3651
UNAfold	0.4155	0.4078*	0.3726*	0.3781**
RNAfold	0.4196	0.4026***	0.3596*	0.3577**
